# Bilingual Sign Language Recognition: A YOLOv11-Based Model for Bangla and English Alphabets

**DOI:** 10.3390/jimaging11050134

**Published:** 2025-04-27

**Authors:** Nawshin Navin, Fahmid Al Farid, Raiyen Z. Rakin, Sadman S. Tanzim, Mashrur Rahman, Shakila Rahman, Jia Uddin, Hezerul Abdul Karim

**Affiliations:** 1 Department of Computer Science, American International University-Bangladesh, Dhaka 1229, Bangladesh; 2 Centre for Image and Vision Computing (CIVC), COE for Artificial Intelligence, Faculty of Artificial Intelligence and Engineering (FAIE), Multimedia University, Cyberjaya 63100, Selangor, Malaysia; 3 AI and Big Data Department, Woosong University, Daejeon 34606, Republic of Korea

**Keywords:** American Sign Language (ASL), Bangla Sign Language (BdSL), deep learning, sign language recognition (SLR), YOLOv11

## Abstract

Communication through sign language effectively helps both hearing- and speaking-impaired individuals connect. However, there are problems with the interlingual communication between Bangla Sign Language (BdSL) and English Sign Language (ASL) due to the absence of a unified system. This study aims to introduce a detection system that incorporates these two sign languages to enhance the flow of communication for those who use these forms of sign language. This study developed and tested a deep learning-based sign-language detection system that can recognize both BdSL and ASL alphabets concurrently in real time. The approach uses a YOLOv11 object detection architecture that has been trained with an open-source dataset on a set of 9556 images containing 64 different letter signs from both languages. Data preprocessing was applied to enhance the performance of the model. Evaluation criteria, including the precision, recall, mAP, and other parameter values were also computed to evaluate the model. The performance analysis of the proposed method shows a precision of 99.12% and average recall rates of 99.63% in 30 epochs. The studies show that the proposed model outperforms the current techniques in sign language recognition (SLR) and can be used in communicating assistive technologies and human–computer interaction systems.

## 1. Introduction

Sign language recognition is among the most thoroughly studied fields in recent years and has many different applications, such as deep learning, human–computer interaction, and pattern recognition [1]. It has significant importance for the future development of human civilization by eliminating the impairment that prevents people with hearing disabilities from communicating effectively with others [2]. It has the social responsibility of facilitating the communication needs of the hearing-impaired, thereby helping to improve society. From 1960, sign language was accepted as a formal language, which is nonverbal and used by the deaf community [3]. As of 2015, there were around 1.33 billion people who experienced hearing loss of some degree and which amounted to 18.5% of the worldwide population [4]. As per the World Health Organization (WHO), in 2024, at least 430 million people will suffer from hearing loss globally and about 34 million of them are children. It is projected that it will reach a population of 700 million or 1 in every 10 by the year 2050 [5]. However, since gestures and signs are diverse and subtle, signs occur at different locations and times, and hand shapes are complex, vision-based sign language recognition is a difficult interdisciplinary field due to difficulties in perception [2]. The signed languages are distinct from the spoken languages but also from each other; today, there are more than 200 signed languages documented. Some of the examples of signed languages are American Sign Language (ASL), British Sign Language (BSL), Bangla Sign Language (BdSL), Japanese Sign Language (JSL), and Chinese Sign Language (CSL), each with distinct grammar and syntax. Therefore, several challenges are experienced by individuals who have disabilities because of limited access to information and communication problems. This restricts the availability of information and communication in a manner that affects the deaf and hard of hearing community, pointing towards the need for an interpreter in the form of a machine translation system that can translate sign language into text or speech [6].

The BdSL, standardized in 2000 by the Centre for Disability in Development (CDD), is the combination of meaningful sequential elements, which are the aspects of hand shape, movement, orientation, and place [3]. This is the reason why the analysis of BdSL is challenging, because 95% of its lexical signs are based on the orientation of both hands. For this reason, the following phonological and morphological characteristics require a good recognition system with effective controlled communication for the deaf. Thus, it is pertinent that a model be developed that may aid the hand gestures and sign language that act as an interaction for deaf and nonverbal people to communicate in society, thus interpreting sign language into text or speech. For establishing such a system, there is a need to come up with a way of categorizing Bangla and English Sign Language.

There are two ways of developing a sign classification: it can be sensor-based or through computer vision techniques. This paper is based on the second one to construct a combined system by employing ASL and BdSL. We present an effective real-time hand gesture recognition scheme using the advanced YOLOv11 for detecting several gestures within a frame that is suitable for real-life scenarios.

The earlier techniques used various hand-categorized features and statistical models for SLR; however, over the past few years, deep learning and convolutional neural network (CNN) technologies have enhanced the SLR performance [7]. Of all the existing object detection architectures, the You Only Look Once (YOLO) series can be considered effective enough for real-time gesture recognition. In YOLO, object detection is posed as a regression problem, and they detect objects and their locations in a single pass through the network. Earlier, YOLOv1 had a frame rate of 45 but less localization than two-stage detectors like Faster R-CNN [8]. However, it has evolved and has usefulness in real-world problems and, in the case of SLR, it has also been used in [9,10]. It uses the concept of division of an image into multiple cells. The image is divided into S×S square grid [11]. Every grid cell provides bounding boxes and confidence scores.

The sign recognition environment begins with the feature extraction phase of ASL and BdSL signs and proceeds with the identification of a proper algorithm for the sign recognition system. Once they have completed the algorithm and trained the network’s architecture, the system is ready for ASL and BdSL sign recognition and classification of 64 distinct alphabets. The contribution of this research work includes the following:

- The system uses sign detection to improve communication between Bangla Sign Language and American Sign Language users by recognizing both Bangla and English letters. First, a custom dataset of 9556 images has been prepared from open source, including different signs of the Bangla and English alphabets. This enhances the generalization performance of the proposed model from earlier related studies.
- We attempted to pretrain on our custom dataset to improve the model’s performance as well as its robustness in various scenarios. We also had to adjust the sizes of the images to the appropriate size and put labels on each of them to provide quality input to enable the training to be done. For preprocessed images, the proposed rapid object detection approach based on the deep learning YOLOv11 model detects the different types of hand gestures with high accuracy in real time.
- A comparative analysis of YOLOv11 has been conducted, and it has also proved that YOLOv11 has better performance than all the basic models, such as YOLOv8, YOLOv9, YOLOv10, and YOLOv12. This shows that the proposed YOLOv11 is better, as found in the evaluation results in all scenarios presented above. The value of evaluation parameters like accuracy, precision, recall, and F1 score was enhanced in comparison to the other recognition techniques.

The layout of the remaining paper entails the following sections: Section 2 presents a comprehensive and comparative synthesis of the literature in this field. Section 3 describes the overall system methodology along with the proposed sign detection framework. This section of the paper presents a summary of the findings and breakdown of the analysis process in Section 4. Finally, the conclusion of the study is presented in Section 5, as well as a proposal for further research.

## 2. Literature Review

General object detection can be categorized into two main types, which are the traditional approach and the deep learning (DL) approach [12]. Historically, object detection was performed with the help of designed features and learning algorithms such as Haar features, HOG features, SIFT features, SVM, and Adaboost [2]. These methods have delivered good performance in certain conditions but, when it comes to complex scenes or different object categories, they fail. With advancements in DL algorithms, the use of deep-learning-based object detection methods has tremendously enhanced. Mainstream object detection based on CNN utilizes the R-CNN series and YOLO series and SDD series with their distinctive advantages and limitations. More specific differences can be recapitulated in the form of Table 1. While emerging in the domain of DL, the general-purpose models include LeNet-5, AlexNet, VGGNet, GoogLeNet, and ResNet in this field, which possess the ability to categorize several target objects and detect their location in the image [2]. In particular, the current target detection system operates through a candidate region, followed by feature extraction, prediction, and non-maximum suppression (NMS), such that four evaluation metrics evaluate network speed through FPS and mean average precision along with the precision–recall curve (RP) and intersection over union (IoU). The metrics presented here enable the evaluation of diverse model performances and the impact of different categories on the model.

Several kinds of variant Sign Language (SL) research, including ASL [13,14,15,16,17,18,19,20,21,22], Indian Sign Language [23], BdSL [24,25,26,27,28,29,30,31], Arabic Sign Language (ArSL) [2], and several others, have been conducted in the past few years. Our first studied research focused on a real-time sign language recognition system of static as well as dynamic gestures [15]. The authors designed a deep learning function for sign detection in real time for hand signs. The aspects of the model were trained on datasets of simple and compound backgrounds and video-based dynamic gestures as well. For their static gesture recognition (2D CNN), they used spatial augmentation techniques to enhance the dataset. Their dynamic gesture recognition (3D CNN) utilized keyframe extraction to simplify the dataset before training and trained it on LSA64. As a result, they achieved 92% accuracy for static signs in a complex background, and the model reached 84% accuracy after training on the LSA64 dataset. The limitation of their research was that the accuracy of the dynamic sign is lower (84%) compared to static recognition.

In the model developed in [16], self-attention mechanism was employed to improve the CNN. It was coupled with a flexing sensor data glove and a gyroscope for tracking movements and capturing significant features of a gesture while filtering unnecessary background noise. It achieved 99.52% accuracy in static ASL gestures in an environment under controlled settings. In standard static gesture recognition tasks, it was outperformed by CNN + BiLSTM (98.21%), BP Neural Network (98.5%), and SVM (99.2%). However, their study only touches the static gestures, and their performance falls off drastically with greater variations in hand positioning. Secondly, this method relies on wearable sensors, which are not feasible in a real-world scenario, and does not have a real real-time deployment strategy. We overcame these problems with our approach. Unlike their approach, we do not need wearable sensors and prepare our dataset with different lighting, hand shapes, and movement variations for better accuracy under diverse conditions.

For recognizing six distinct hand gestures in SL, the study presents a hand feature-based method that utilizes convexity defects for gesture classification and a deep-learning-based YOLOv3 model with DARKNET-53 CNN as the backbone [17]. For training the model, transfer learning was performed using DARKNET-53 on a large custom dataset with the help of non-maximum suppression for gesture recognition. Their results are 95.57% for the first approach under controlled conditions, and the second approach based on deep learning has 98.92%. However, the drawback here is it covers only six static gestures in ASL, and its hand feature method is not as proper for real-life problems with the sensitivity issue in lighting and noise.

A hybrid model CNN with spatial pyramid pooling (SPP) was introduced to improve hand gesture recognition in vision-based systems [18]. The CNN-SPP model applied two convolutional blocks, containing four convolutional layers in each block. To solve the problem of fixed-length feature representation irrespective of the size of the input image, we integrated SPP after each convolutional block. It uses nine types of data augmentation like shear, gamma correction, salt and pepper noise, perspective transformation, and others. The model used five-fold cross-validation for performance assessment for evolution and testing. As a result, the SPP significantly improved feature extraction, reducing feature map size by 80% while preserving accuracy. The limitation was that this model does not properly address sequences of gestures in a manner that guarantees real-time deployment.

A machine-learning-based SL recognition system that is optimized for low computational power devices was also proposed [19]. The system extracts key features from images rather than processing entire images, making it suitable for real-time mobile applications. The feature extraction techniques help them in minimizing the computational load. It implemented a client–server architecture for recognition. Their result shows that the system yields a 90.12% success rate for the alphabet of ASL recognition.

In the study [20], a deep-learning-based solution for SLR from digital videos is proposed using a Detection Transformer (DETR) with Feature Pyramid Network (FPN). They aimed to enhance the accuracy of recognition by using ResNet152 as a backbone and FPN for multi-scale feature extraction. They also compared ResNet152 + FPN + DETR and YOLO-based models, respectively. From there, they achieved almost 96.45% accuracy for the RNN model, as they outperformed other models. Introducing ResNet152 + FPN + DETR also enhances the accuracy of SLR by 1.7% compared to standard DETR, plus inference is faster than 28 FPS, which is suitable for real time. But the limitation of that model is that it recognizes only nine signs and no multilingual support and, most importantly, for the DETR-based approach, we need to use a powerful GPU (RTX 3060) for efficient training and inference.

An ASL alphabet recognition system based on hand pose estimation utilizing MediaPipe Hand API to extract 21 hand joint co-ordinates from RGB webcam images computed distance-based features and angle-based features, employing Support Vector Machine (SVM) and Light Gradient Boosting Machine (LightGBM) classifiers [21]. Grid search was used to tune hyperparameters for SVM and LightGBM. The system achieved 62 FPS on a standard laptop. Accuracy of 99.39% on the “Massy Dataset”, 98.45% on the “Finger Spelling a Dataset”, and 87.6% on “ASL Alphabet Dataset” was obtained, where LightGBM performed slightly worse than SVM in all datasets.

Using a Leap Motion Controller, an ASL learning application, a Whack-a-Mole-style game was designed, where users must correctly sign ASL letters to progress [22]. In this paper, LSTM and RNN are employed for the task of classification, and kNN is also incorporated. And, for validation, the 5-fold cross-validation is used. To train the model, the authors tested different batch sizes of (32, 64), LSTM units of (28, 30), and epoch values of (30, 40, 80) using categorical cross-entropy loss and Adam optimizer. From there, 5-fold cross-validation was adopted to obtain a 91.82% accuracy. In terms of recognition, the LSTM-RNN model recorded a better performance and was better than the traditional SVM and RNN models. However, the model described above is made only for right-hand gestures and cannot be generalized to the whole extremities.

In [24], CNN-LSTM was applied for BdSL recognition based on lexical signs. The authors noted that existing ML-based approaches like SVM, PCA, kNN, and ANN have limitations of over-fitting and BAD GC when a large dataset is used; to overcome that problem, the CNN-LSTM model has been put forward. For their CNN layers, they used two convolutional layers, two max pooling layers, and two dropout layers (25% neurons deactivated to prevent overfitting). It was trained with Stochastic Gradient Descent (SGD) and with learning rate = 0.001 and Nesterov for optimization purposes and had usually faster convergence. The CNN-LSTM reported 90% on training and 88.5% on testing on the BdSL dataset, as reported by the authors. VGG16 has the problem of accuracy fluctuation, with testing accuracy ranging between 65% and 70%. The disadvantage of that model is that it is static. The training accuracy was slightly higher than testing accuracy (88.5%), indicating a minor overfitting issue as well, and the system was tested only on a high-performance computing setup without optimization for embedded systems.

In the interest of establishing a low-cost and real-time Bangla sign recognition solution that does not depend on cloud resources, an edge computing device, namely NVIDIA Jetson Nano, was introduced [25]. Detectron2 (Faster R-CNN with FPN) and EfficientDet-D0 with BiFPN and YOLOv7Tiny models were used for training and Jetson Nano for real-time evaluation. From the results found, Detectron2 offered the highest accuracy of 94.91% mAP@0.5; however, its usability on the edge was inhibited by high computational requirements. While the average precision for objects at 0.5 IoU was impressive, 94.0% for EfficientDet-D0, it took longer for the model to train. YOLOv7 Tiny provided the best real-time performance, reducing training time by 60% compared to EfficientDet and 63.25% compared to Detectron2, and an average FPS of 24 was achieved on Jetson Nano for real-time detection. But no support for continuous gesture recognition, limited vocabulary, and no multilingual support are the limitations of this model.

In [26], a novel architecture for recognizing BdSL combining transfer learning-based CNN and RF is presented. It detects BdSL numerals from images and distinguishes between alphabets using pretrained architectures (VGG16, VGG19, InceptionV3, Xception, and ResNet50) and classifies signs using Random Forest. Fully connected layers are transmitted with a Random Forest classifier, employed SGD as a classifier, and Categorical Cross-Entropy for losses. The performance of the model was evaluated using accuracy, precision, recall, F1 score, and ROC curves. This method has 91.67% accuracy, 93.64% precision, 91.67% recall, and 91.47% F1 score in character recognition.

To develop a computer vision-based machine translator, a CNN-based model for recognizing BdSL digits was proposed [27]. In their CNN architecture, they used convolutional layers to extract the hand shape feature. After the training, the accuracy was 95.35%. Only supporting static digit recognition, not being optimized for real-time deployment, and no transfer-based feature extraction are the major limitations of the model.

Integrating spatial, skeletal, and edge-based image features, a multi-model deep learning approach for BdSL recognition, a three-stream CNN model combined with nine ensemble meta-learning algorithms to improve hand pattern recognition accuracy was proposed [28]. For preprocessing, they have used spatial feature extraction, skeletal feature extraction, and edge feature extraction. The model is evaluated on training loss, accuracy, recall, precision, and F1 score for different architectures. From the outcome, it reflects 99.77% training accuracy for spatial CNN, 98.11% training accuracy for skeletal CNN, and 99.3% in edge-based CNN training. ResNet50 performed best among pretrained models (99.87% accuracy) but required high computational power.

For continuous recognition, the MVBSL-W50 model was introduced with an attention-based Bi-GRU model that focuses on movement patterns while disregarding body appearance and background noise [29]. Attention mechanism helps focus on key sign patterns, improving recognition. Bi-GRU with attention achieved the highest accuracy of 85.64%, which was quite desirable and higher than other RNN variants. However, the key issue associated with the model is that the model is not efficient for real-time applications and has gesture-similarity problems. It also has MediaPipe limitations, and it failed to detect two-handed gestures effectively.

In [30], a real-time bidirectional 3D Bangla Sign Language (BdSL) translator that automatically generates animated BdSL gestures from Bangla text and voice inputs was introduced. It uses Signing Gesture Markup Language (SiGML) and Hamburg Notation System (HamNoSys) to realistically translate Bangla numerals (0–9, Thousand, Lakh, Crore) to 3D animated sign language. The architecture takes voice input using Speech-to-Text (STT) and text input using Text Parsing and performs the model’s tokenization, then maps the input to the correct BdSL sequence. From their findings, they were able to determine that the new proposed model for detecting gestures had a shorter time of 6.5 milliseconds, while established models of rule-based systems took 9.5 milliseconds.

BSL recognition, namely the Bangla Language Modeling Algorithm (BLMA) system, has two stages: first, hand-sign classification using Normalized Outer Boundary Vector (NOBV) and Window-Grid Vector (WGV) for BdSL letters [31]. Classification of ambiguous signs is conducted using the second classifier with Decision Threshold (THICC = 0.85). Then, in the second phase, it targets to make full words, numeral, and sentence identification with the help of detecting hidden characters. Once the training and evolution phase was completed, it was possible to obtain a mean accuracy of 95.83% for hand-sign classification in the first phase of the system. BLMA yielded 93.50%-word recognition, 95.50% numeral recognition, and 90.50% full-sentence recognition; the computational cost was 39.97 milliseconds per frame; hence, real-time recognition was possible. The limitation of the system was that it only relied on traditional features like NOBV and WGV rather than deep learning features like CNN or transformers.

## 3. Materials and Methods

In the following sub-section, we have described the research implementation procedure in detail. Our proposed method enhances a deep Convolutional Neural Network (CNN) structure that is solely developed for identifying BdSL and ASLs. In Section 3.1, a flowchart presents a simple approach for how BdSL and ASL can be identified from images as described.

### 3.1. Workflow Diagram

**Figure 1:**
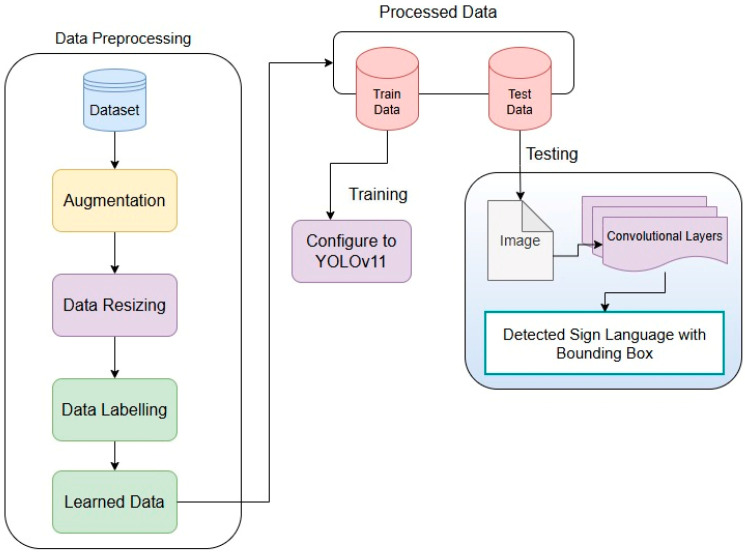
Workflow diagram for the model.

For a better analysis of this paper’s concept, a workflow diagram for the whole approach to detect the sign is presented in Figure 1.

### 3.2. Image Acquisition and Dataset Preparation

**Figure 2:**
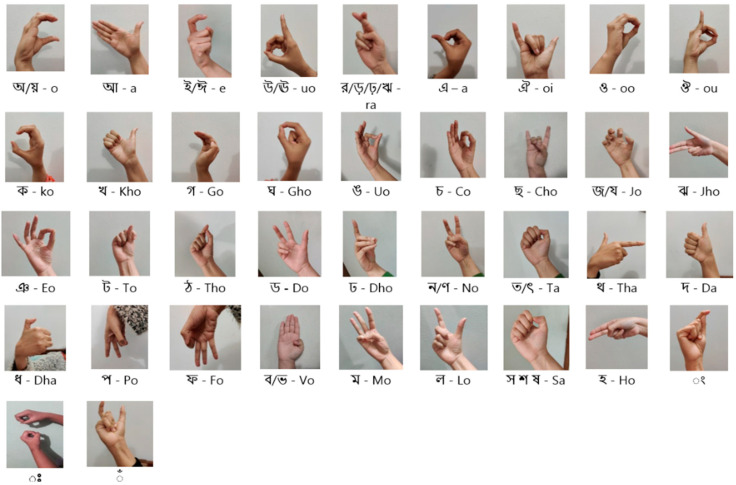
BdSL representation for Bangla alphabet.

**Figure 3:**
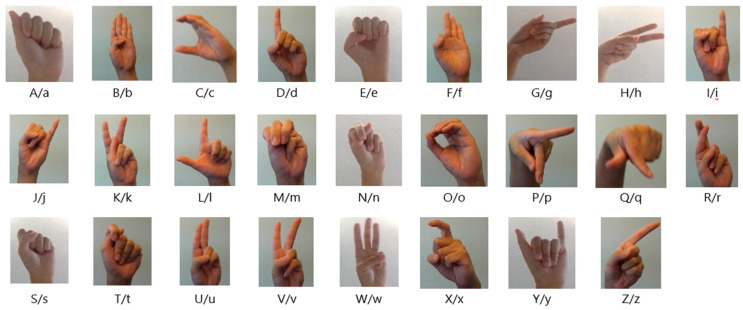
ASL representation for the English alphabet.

Image acquisition is the first process involved in the creation of an efficient sign language detection framework. After collecting the image data, the next step is dataset preparation. For this study, the dataset contains 9556 images, each representing one of the 64 unique letter signs in various signs in Bangla and English sign language, which were collected from online reliable sources. The BdSL dataset was collected from the “BdSL 47_dataset” [32], and the ASL dataset was collected from the “ASL Signs” dataset from Kaggle. Data annotation, data preprocessing, and data augmentation are all included in this subsection for preparing the dataset. To increase the generalization ability of the output, the hand gestures differ in variability and complexity, which are present in the dataset. Figure 2 and Figure 3 show sample image datasets for BdSL and ASL.

### 3.3. Data Augmentation

**Figure 4:**
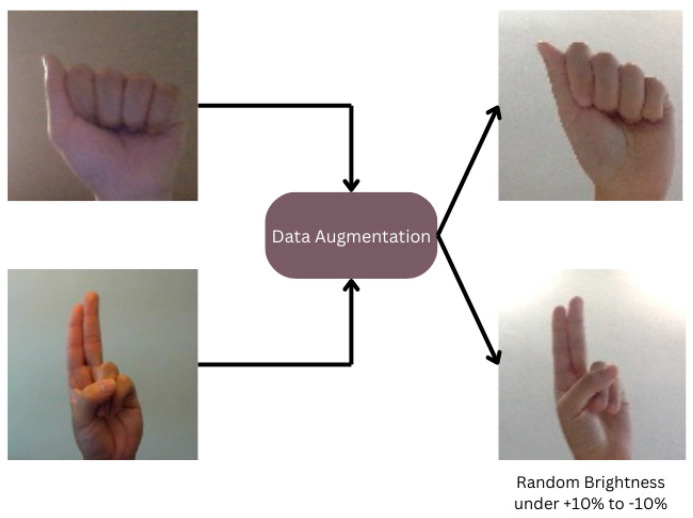
Sample data augmentation.

There could be overfitting of the models if the data collected are not enough and are of a small size. Data augmentation schemes are used to overcome this problem since; besides the existing data volume, more data need to be included. Data augmentation is used to obtain more varied and diverse variants of a given set to expand the range of possible applications of one or more ML/AI models. We used Roboflow as a platform that allows making deeper work with deep learning tools easier and faster. The tool supports data labeling, preparation, model training, and even final model deployment in the target environment. For augmentation, several techniques, like crop and rotational augmentation techniques, are applied. As our dataset is collected from various sources, in some cases, we need to crop the data and rotate some of them and label them as shapes. This method involves scaling some of them by randomly applying rotations of random angles between −10 degrees and +10 degrees [33]. This augmentation technique is to simulate a real-world setting that can result in objects appearing in any orientation to increase the variety of the dataset. Training the model through rotated images enables the model to memorize and generalize when it is exposed to rotated real images during the deduction or testing phase, giving better performance. Following this, there were some images in the dataset with low brightness, and this was the reason we had to apply the brightness correction. In our procedure for the data augmentation step, we made some random changes in the brightness levels. On a percentage scale, the adjustment degree ranged from +10% to −10% [34], which is presented in Figure 4. This method proved instrumental in improving the visibility and definition of dimly lit images, rendering them more conducive for analysis and facilitating their suitability for model training purposes [35]. Also, we selected some random data and then just flipped it into a different position. We used this technique to enhance our dataset.

### 3.4. Data Preprocessing and Labeling

**Figure 5:**
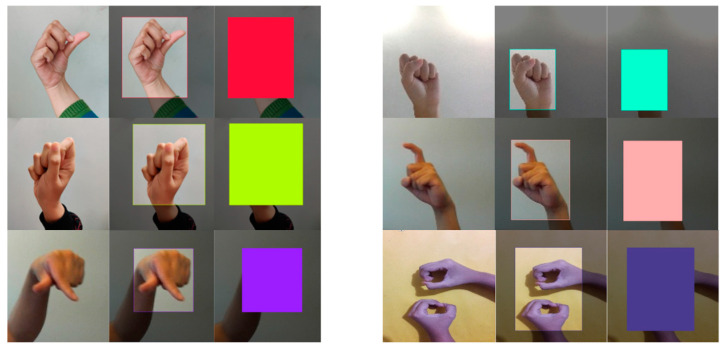
Sample labeled data.

After data annotation, the next part is data preprocessing. Preprocessing is one of the most efficient ways of enhancing the quality of images and their preparation for analysis. Some of the common image preprocessing techniques include noise reduction, contrast enhancement, and resizing [36]. We applied the auto-orient, resized the image to 640 × 640, and made contrast adjustments for image preprocessing. The 64 classes displayed in Table 2 were used to label the resized images. The annotation tool of Roboflow was used to label data into multiple classes. In Figure 5, the first column shows the raw input images, the second column contains the class, which is labeled using a square bounded box, and the third column shows the result when the bounded box layer is on, differentiating classes with different colors. In this section, the 64 class IDs are represented just like the following Table 2. For example, when the model detects the class “English_G”, it represents the English alphabet “G” and, if the model detects the class name “Bangla-8”, it represents the Bangla alphabet “ও (oo)” and this method will apply for all the other classes, which is provided in the Table 2 dataset statistics.

All the steps for the data preprocessing is displayed in a flowchart to understand the architecture of the model better in Section 3.5. The collected dataset was further divided into training, validation, and testing datasets in 70%, 15%, and 15%, respectively, for better evaluation of the model. Since, in machine vision-based deep learning models, there is a requirement for many images, the data size is inflated using the data augmentation technique.

### 3.5. Proposed Sign Detection Framework

**Figure 6:**
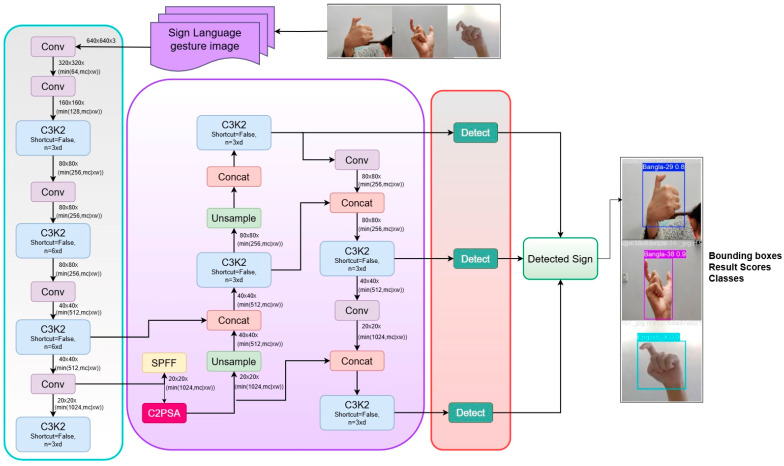
Proposed YOLOv11-based architecture.

Our sign detection framework utilizes the YOLOv11 algorithm, a state-of-the-art object model. YOLOv11 is an improved version of the YOLO model family that is specifically used in real-time object detection. The YOLOv11 algorithm is one of the newest algorithms in the YOLO series, which was improved based on the YOLOv10 algorithm. YOLOv11 can obtain accurate, real-time, and efficient detection results. Compared with YOLOv10, YOLOv11 realizes model light-weighting under the premise of guaranteeing accuracy, and the YOLOv11 network has high detection accuracy and fast inference speed up to 50 ms, with the fastest detection speed compared to any other YOLO version [2]. Among the enhancements made in YOLOv11 is an upgraded backbone network for better feature extraction. In enhancing accuracy, new optimization of anchor boxes along with the new and advanced loss functions are utilized. It focuses on the areas that are most crucial for an image and, through a new path aggregation network and with the help of transformer-based attention, boosts the multi-scale feature fusion. The architecture of YOLOv11 in Figure 6 is a contemporary deep learning model for real-time object detection. This model is used to enhance the consideration level and the time complexity to find the best suitable method of English and Bangla Sign Language detection. The model begins with an input image of a hand sign as a Bangla or English sign language gesture.

Next, the feature extraction begins, and these are standard convolutional preparations and start extracting features from the given image inputs. To perform the operations on the input image and to reduce the spatial size of the feature map and increase the depth, multiple convolutional layers are introduced. Also, to obtain deep feature extraction, such complex convolutional layers are included. Shortcut = False and n = 3x or n = 6x are configurations of the layers to be used in this case. Concatenation feature maps are present to accomplish the fusion of information from different levels of the network at different points. In the C2PSA module, spatial information in feature maps that integrate multi-scale features are applied. It is used before the data is forwarded to the detection layers. The detection layers generate the location boxes and the likelihoods for specific objects via final convolutions and then combine them with some others. The output from the model highlights the detected results with bounding boxes and labels placed on the images to show which sign language it relates to.

## 4. Results

This section covers an experimental analysis of the YOLOv11 model discussed in the current study and compares it with other models, as well as other YOLO models with varying parameters and some of the existing models.

### 4.1. Hyperparameters

The YOLOv11-based model training encompasses several training settings and hyperparameters for the real-time identification and detection of sign language. This section outlines the details of the hyperparameters that were used in the training process of the model. Table 3 provides a list of the parameters used for the algorithm and the values assigned to each of them. For the training step, the epoch is 30, the optimizer is selected as an SGD optimizer, and the pretrained model is taken as a model pretrained on the COCO dataset. To avoid overfitting when training the present model in the process of building, an early stopping is incorporated into the training process to avoid overtraining the model. Training is interrupted when no improvements are made in the previous 15 epochs.

Other parameters include the batch size, the learning rate, and the weight decay, which undergo standard configuration for the enhancement of the model. A batch size of 16 was chosen because increasing the batch size beyond 16 would significantly slow down the machine learning process and lowering the batch size below 16 did not lead to improved model performance. While larger batches help to converge faster and reduce the noise in gradients, they also increase the GPU memory consumption. On the other hand, smaller batches drastically increase the size of gradients but result in noisy gradients. The Stochastic Gradient Descent (SGD) optimizer was used for optimizing the model due to its efficiency while dealing with large data and its ability to converge. The learning rate was set to be 0.01 due to a preliminary search, as it was found to be balanced between convergence and overshooting. Weight decay of 0.0005 was used to protect against overfitting since large weights violate the laws of both common sense and data interpretation.

### 4.2. Model Evaluation

From the model evaluation, a high level of accuracy was achieved and observed in the trained YOLOv11s model for sign language detection. Table 4 represents the model parameters. About the software environment of the model evaluation, this study was conducted on Ultralytics 8.3.40 Python-3.11.11 torch-2.5.1+cu124 with CUDA:0 using Google collab Tesla T4 plan that has a System Ram 12.67 GB and Disk 112.64 GB. This model, containing 238 layers, gradients = 0, and 9,456,896 parameters, displayed a high level of computational functionality, attaining a GFLOPs of 22.6. Evaluation and metrics that are part of the evaluation process further contribute to the efficiency of the model in the sign language sector.

### 4.3. Result Analysis

**Figure 7:**
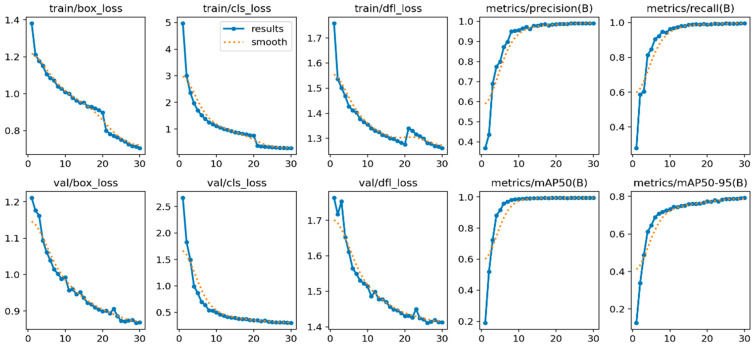
YOLOv11-based training results graph with 30 epochs.

**Figure 8:**
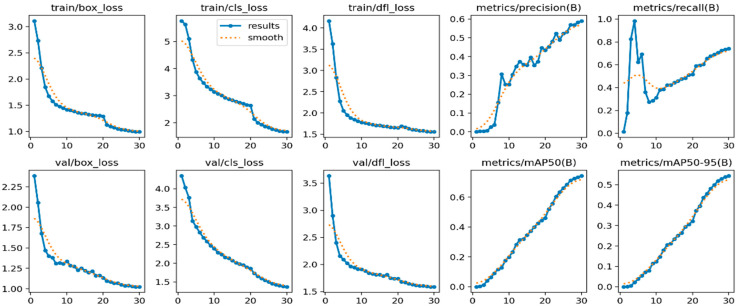
YOLOv12-based training results graph with 30 epochs.

**Figure 9:**
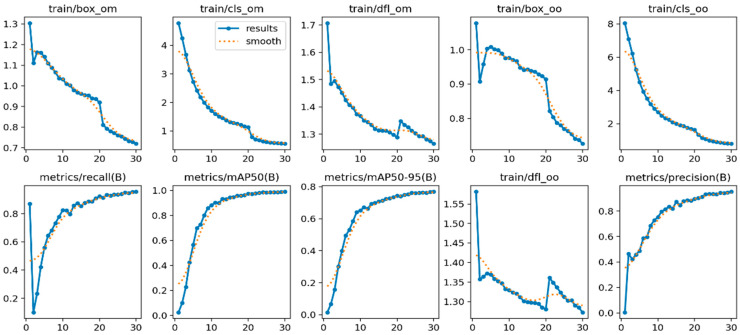
YOLOv10-based training results graph with 30 epochs.

From the visualized training results shown in the results.csv file and the training result images, it can be noted that the dataset provides excellent training performance. Training graphs using YOLOv11 are presented in Figure 7, Figure 8 and Figure 9, where YOLOv12 and YOLOv10 graphs for comparison are also shown. Moreover, the YOLOv11, as proposed in this study, was found to have a recall of 99.63%, a precision of 99.12%, and a mAP of 99.4% when it was trained for 30 epochs.

In Figure 7, the row training loss matrices reveal that train/box_loss improves from 1.4 to 0.8, suggesting that bounding box predictions are improving. Train/cls_loss reduces from 5 to slightly above 0, indicating better prediction of the object categories in the box. Reduction in train/dfl_loss from 1.7 to 1.3 results in better model performance on this composite loss parameter. Based on the performance measurements, when the metrics/precision(B) is between 0.4 and slightly over 1, metrics/recall(B) is between 0.4 and almost 1, mAP matrices/mAP50(B) is between 0.2 and 0.8, and mAP50-95(B) is between 0.2 and 0.6, the model can fully and accurately predict class “B”. The outcomes depict that the model tends to identify detections having augmented ground truth overlap at numerous thresholds while involving validation loss at faithful detection levels.

On our custom dataset, we test the object detection model presented in this paper, called YOLOv11, and other models such as YOLOv8, YOLOv9, and YOLOv10, and the latest version of it, YOLOv12. Table 5 shows an instance of various models detailing how they performed in all the classifications with different steps of iterations. It evaluates the performance of the detector by mAP@0.5, which depicts the mean average precision in the training phase, during wich the detector learns with the keys of the set with validation data. Here, a value giving a higher result suggests better learning. After that, the F1 score is calculated from the following equation:

$$F1score=\frac{\left(2\times Precision\times Recall\right)}{\left(Precision+Recall\right)}$$

In general, the proposed model has a high accuracy based on the F1 score of 0.994 and mAP@0.5 of 0.99. Table 5 displays the model complexities for all models. F1 score and mAP@0.5 are higher than other YOLO-based models’ data visualization because of detects single classes in a portion of the sign language detection dataset, capturing subsequent unique single images. On the other hand, the proposed YOLOv11 receives significantly higher results than other models, as shown in Table 5 below. Because of its higher number of trainable parameters, the YOLOv11 model is the one with the most generalizability among all the models.

Further, the precision of the prediction rate can also be seen in the confusion matrix diagram, which is illustrated in Section 4.4.

### 4.4. Visualization

**Figure 10:**
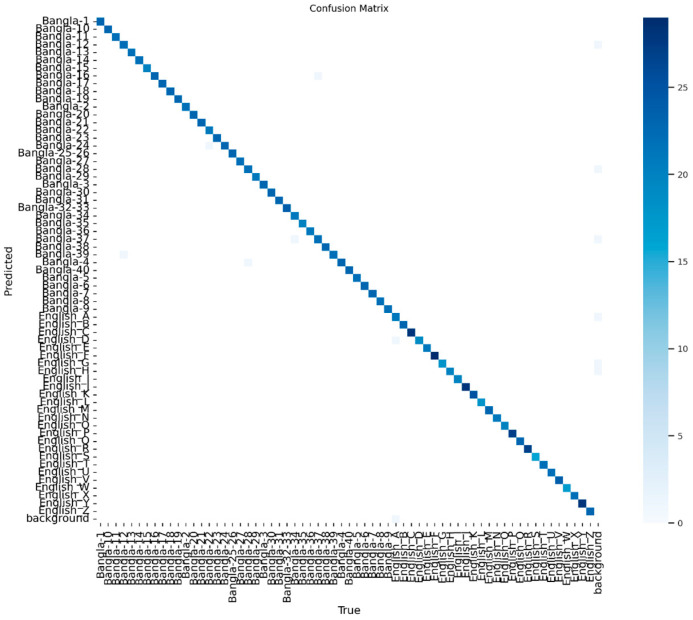
Confusion matrix of proposed YOLOv11-based model results for 30 epochs.

**Figure 11:**
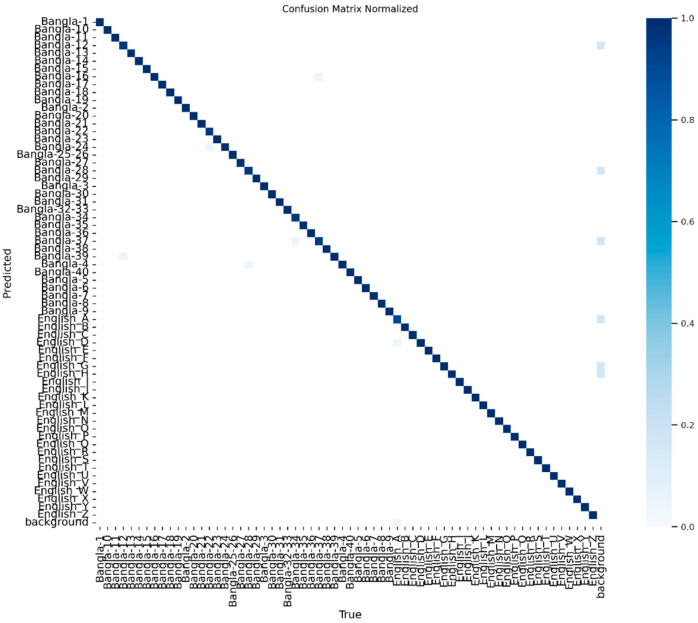
Confusion matrix of proposed YOLOv11-based model detection results after data normaization.

The example training is completed in 30 training cycles or epochs. The term epoch refers to one pass of the whole dataset through the neural network. When training a model, its parameters are adjusted in parallel with the determined loss, as well as the gradients. The precision of the prediction rate is represented with the confusion matrix diagram, which is illustrated in Figure 10 for 30 epochs. Figure 10 illustrates the confusion matrix of the proposed model. Figure 11 shows the confusion matrix of the dataset after normalization and, similarly, as seen here, the miss detection rate was greatly reduced after conducting the normalization. The proposed model training process is completed after approximately 1 h 30 min. They can vary depending on the available computational power and the hardware to be employed within the learning process.

**Figure 12:**
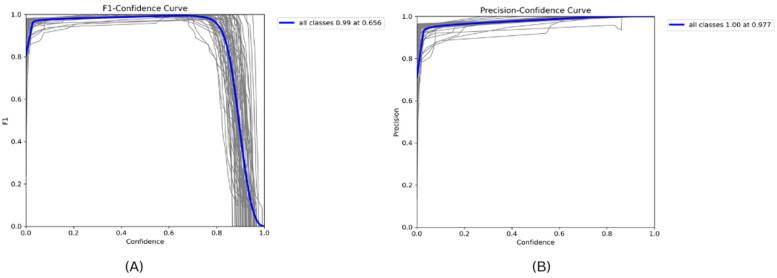
ASL-BdSL sign detection: (**A**) F1–confidence curve and (**B**) precision–confidence curve for proposed YOLOv11-based detection model.

In Figure 12A below, the horizontal axis shows the confidence level, and the vertical axis shows the F1 score. The vertical blue line labeled by “all classes 0.99 at 0.656” indicates that, when the model is allowed to make modal predictions above a confidence score of around 0.656, the F score for all classes combined is nearly 0.99. This high value means that the model has a good accuracy rate and performs well. In Figure 12B, the value depicts the confidence threshold, estimated by the model for the probability of forecast accuracy. The *y*-axis represents the model’s accuracy at the different confidence levels. The “all classes 1.00 at 0.977” bold blue line indicates the accuracy of the proposed model is 1.00 or 100% for all classes when predicting 100 percent of the actualities. This model is appropriate as it gives a definite probability estimate that is reliable.

**Figure 13:**
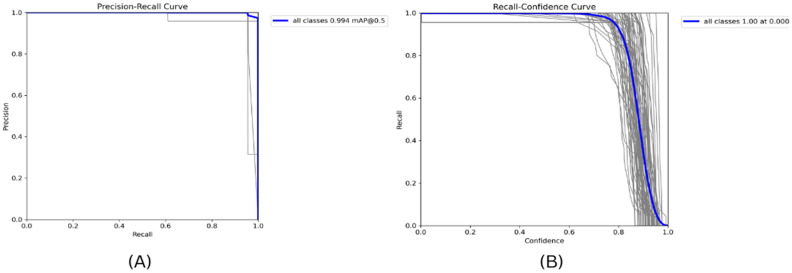
(**A**) Precision–recall and (**B**) recall–confidence curve for the proposed YOLOv11-based model’s ASL-BdSL identification method.

In the experiment presented in Figure 13A, there are different experiments and iterations to fine-tune the precision and recall being typically close to the upper-right corner of the graph [10]. The different gray lines in the figure represent the trade-off between accuracy and recall of different classes or runs of the model. The blue line labeled as “all classes 0.994 map@0.5” highlights the mAP and that the model reaches a 0.994 mAP level using the IoU threshold, where the model is tested with a value of 0.5, which is commonly used in object detection problems. The mAP value is high and indicates that, at this threshold, a good rate of precision and recall for all classes is achieved when using the proposed model.

**Figure 14:**
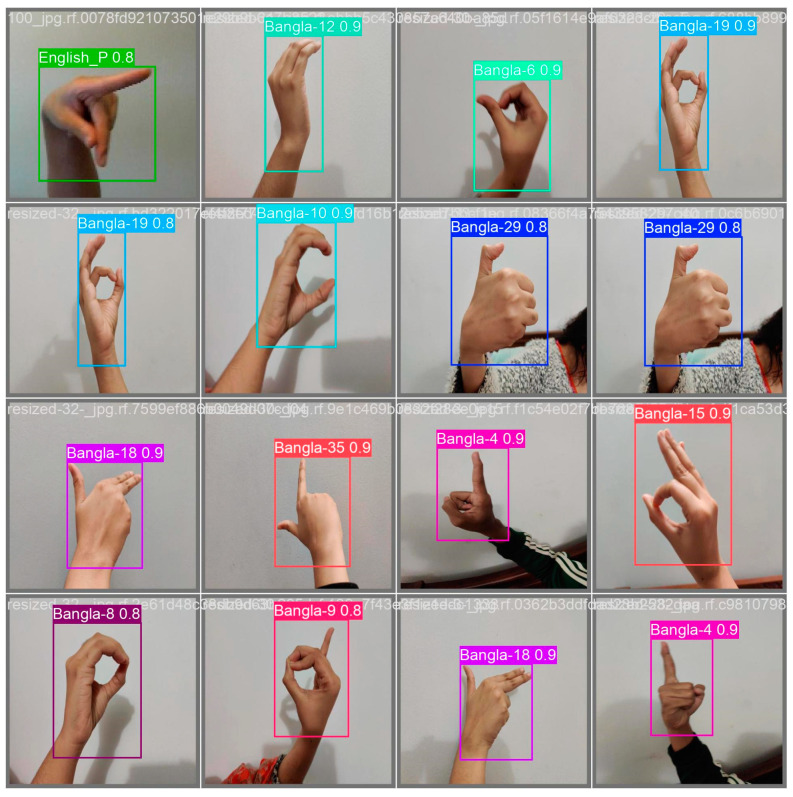
Testing performance for random data.

Figure 13B demonstrates the efficiency of the method used to identify ASL and BdSL, together with a recall–confidence model that further establishes the viability of the model to make as many observations as possible in every category. The *x*-axis provides a confidence level. As mentioned earlier, it is confirmed that the proposed model is accurate in terms of ASL and BdSL detection. With the help of fine-tuning and parameter settings, we have reached the maximum recall–confidence score of 100%. Therefore, the method at hand is deemed to be the most suitable in classifying BdSL and ASL gesture images at that certain level of confidence. By observing this dense blue line with the label “all classes 1.00 at 0.000”, we can see one of the factors that makes our model highly effective: this single line constitutes the entire recall, which means that all the classes are easily recalled. On the other hand, the gray lines mean different kinds or types, depending on the context, as several lines that are gray and of different lengths and positions are observable. These symbols imply that the recall of the model in response to increasing or decreasing confidence levels is dynamic, and the performance of the model illustrates that it can identify all instances relevant to the model at a confidence level of 0. This curve is useful to interpret how much recall can be obtained for this class and the confidence level of predictions, which is important for optimizing the models’ performance in cases with the accurate detection of BdSL and ASL. Figure 14 depicts the number of classes obtained for some random data, where only one class is detected.

The outcome of the proposed model with the different signs is displayed in Figure 14. According to the above analysis, the correctness of the model is higher in most cases than the detection model. The following experimental results indicate that the proposed detection model has high accuracy, precision, and can also recall more important objects, as shown in the lowest training and validation loss.

**Figure 15:**
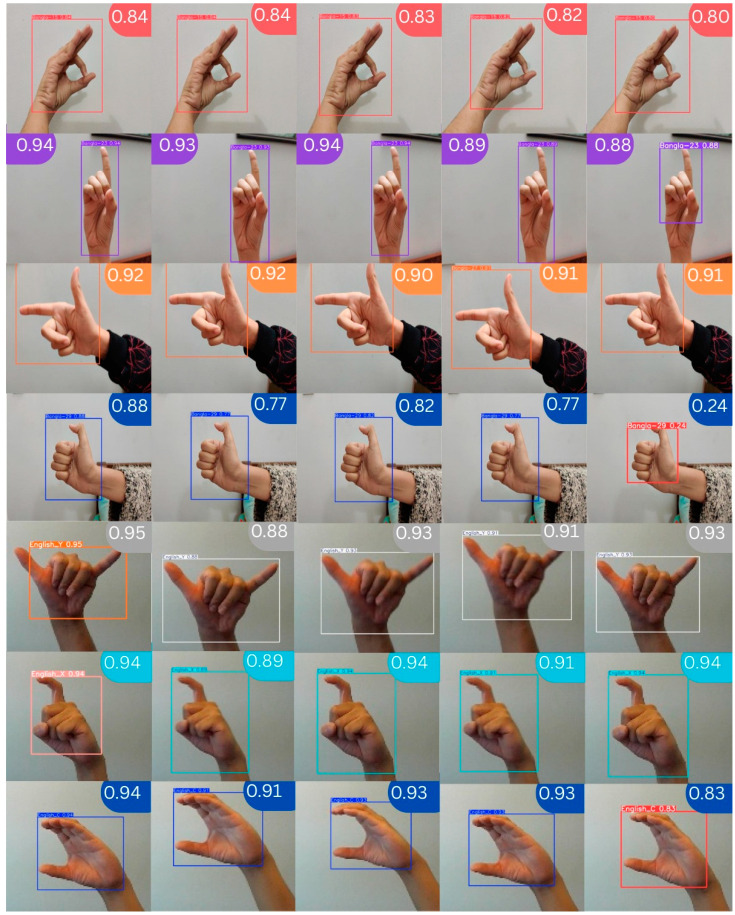
Sample detected images for YOLOv11, YOLOv12, YOLOv10, YOLOv9, and YOLOv8, respectively.

The corresponding individual detection accuracies of all the classes for training the detection steps of YOLOv11, YOLOv12, YOLOv10, YOLOv9, and YOLOv8 for 30 epochs are depicted in the following Figure 15. For BdSL, concerning the class Bangla-15 identified as “চ (cha)”, the scope of Figure 15 reveals that the detection rates stand at 84%, 84%, 83%, 82%, and 80% for the respective versions: YOLOv11, YOLOv12, YOLOv10, YOLOv9, and YOLOv8. Likewise, for the Bangla-23 as “ঢ (Dho)” class, the detection rates were found to be 94%, 93%, 94%, 89%, and 88%. For the Bangla 27 as “থ (Tha)” class, they are 90%, 89%, 89%, 91%, and 89%, respectively. For Bang-la-29 as “ধ (Dh)” class, they are 88%, 77%, 82%, 77%, and 24% for the YOLOv11, YOLOv12, YOLOv10, YOLOv9, and YOLOv8, respectively. When it comes to ASL for the class English_Y, it has been established as 95%, 88%, 93%, 91%, and 93%. In the class English_X, the percentage level achieved is at 94%, 89%, 94%, 91%, and 94%. Hence, for the class English_C, we have achieved the following scores on YOLOv11, YOLOv12, YOLOv10, YOLOv9, and YOLOv8: 94%, 91%, 93%, 93%, and 83%, respectively. Therefore, for all classes, in most cases, the proposal of the sign detection model, YOLOv11, achieves better detection accuracy compared with the other models, including YOLOv12, YOLOv10, YOLOv9, and YOLOv8 with 30 epochs.

## 5. Conclusions

For this study, an approach called the YOLOv11 model is introduced for identifying the Bangla and English sign languages in different settings. This work is aimed at enhancing the effectiveness of the function related to sign detection since it diversifies methods and approaches involved in detecting both Bangla and English sign alphabets. For the proposed research, a new dataset, which consists of 9116 images, was compiled, which combines Bangla and English alphabets, which are also portrayed in sign language. It also contributes to increasing the generalization abilities of the model compared with the previous work. Annotation of the custom dataset and data augmentation were applied to enhance the model’s performance during training and across multiple environments. All the images were labeled thoroughly to feed the model with quality data. For the object detection, the YOLOv11 model was trained for 30 epochs, and the results that we obtain are recall 99.63%, precision 99.12%, F1 score 99.37%, and mean average precision (mAP) 99.4%. From the above-mentioned detection results, the YOLOv11 has outperformed other baseline models (YOLOv8, YOLOv9, YOLOv10, and the most recent YOLOv12). The findings confirm the effectiveness of the proposed model in the identification of Bangla and English alphabets’ sign language without the need for extensive training, which can be used as a standard reference for real-time applications. The research relates to the feasibility of assistive technology for the hearing- and speech-impaired, service delivery areas such as hospitals or self-service kiosks for helping the especially abled, who rely on sign language, and eliminating the barriers of communication, and, lastly, in augmented reality and translating into text or voice. Apart from its linguistic application for the integration of these two sign languages as a way of accommodating those from two different linguistic backgrounds, it also applies to games, virtual reality, and other interactive systems.

However, certain difficulties exist in terms of optimizing the system over environmental conditions and benefiting the possible population. Future work will necessarily involve the collection of a greater variety of gestures and combine them with emotional state detections so that the system can recommend uplifting content to enhance the user’s experience and engagement, as well as focusing on the refinement of the algorithms applied in the experiment.

## Figures and Tables

**Table 1:** Detection methods in object detection [2].

Algorithm Type Contrast	R-CNN	YOLO
Types of detectors	Two-stage	Single-stage
Velocity (FPS)	Lower than the single-stage	Higher than the two-stage
Average accuracy	Higher than the single-stage	Lower than the two-stage

**Table 2:** Dataset statistics.

Class Name	Represented Alphabet	Dataset Quantity	Total
Bangla-30	প	153	
Bangla-31	ফ	153	
Bangla-32-33	ব/ভ	152	
Bangla-5	র/ড়/ঢ়/ঋ	152	
Bangla-13	ঘ	151	
Bangla-12	গ	151	
Bangla-1	অ/য়	151	
Bangla-8	ও	151	
Bangla-4	উ/ঊ	151	
English_G	G	151	
Bangla-23	ঢ	150	
Bangla-27	থ	150	
English_Z	Z	150	
Bangla-39	ং	150	
Bangla-18	ঝ	150	
English_U	U	150	
English_O	O	150	
English_M	M	150	
Bangla-37	হ	150	
Bangla-10	ক	150	
English_L	L	150	
English_B	B	150	
English_F	F	150	
Bangla-40	ঃ	150	
Bangla-14	ঙ	150	
English_X	X	150	
English_A	A	150	
Bangla-3	ই/ঈ	150	
Bangla-19	ঞ	150	
Bangla-28	দ	150	
Bangla-38	ঁ	150	
Bangla-16	ছ	150	
English_R	R	150	
English_C	C	150	
Bangla-25-26	ত/ৎ	150	
English_Y	Y	150	
English_Q	Q	150	
Bangla-7	ঐ	150	
Bangla-24	ন/ণ	150	
English_E	E	150	
English_I	I	150	
Bangla-2	আ	150	
Bangla-20	ট	150	
Bangla-17	জ/য	150	
Bangla-11	খ	150	
Bangla-6	এ	150	
English_J	J	150	
English_S	S	150	
English_P	P	150	
Bangla-21	ঠ	150	
English_V	V	150	
English_K	K	150	
English_D	D	150	
English_W	W	149	
English_N	N	149	
Bangla-22	ড	149	
English_T	T	149	
English_H	H	149	
Bangla-9	ঔ	146	
Bangla-29	ধ	143	
Bangla-36	স/শ/ষ	140	
Bangla-15	চ	135	
Bangla-35	ল	130	
Bangla-34	ম	108	

**Table 3:** Parameters used for the YOLOv11-based object detection model.

Parameters	Value
Batch size	16
Epochs number	30
Optimizer	Auto
Pretrained	COCO Model
Pretrained	0.01
Weight decay	0.0005
patience	100

**Table 4:** Model parameters.

Parameters	Value
Batch size	319
Model parameters	9,456,896
Layers	238
GFLOPs	22.6

**Table 5:** Testing performance of YOLOv11, YOLOv12, YOLOv10, YOLOv9, and YOLOv8.

Model	Epoch	Class	Trainable Parameters	F1Score	mAP@0.5
Proposed YOLOv11	30	All	9.46 M	0.994	0.994
Proposed YOLOv11	15	All	9.46 M	0.991	0.994
YOLOv12n	30	All	2.58 M	0.657	0.743
YOLOv12n	15	All	2.58 M	0.458	0.499
YOLOv10 [37]	30	All	2.73 M	0.954	0.990
YOLOv10 [37]	15	All	2.73 M	0.888	0.961
YOLOv9 [38]	30	All	1.98 M	0.986	0.993
YOLOv9 [38]	15	All	1.98 M	0.966	0.989
YOLOv8 [37,39]	30	All	11.17 M	0.993	0.786
YOLOv8 [37,39]	15	All	11.17 M	0.991	0.994

## Data Availability

The data presented in this study are available on request from the corresponding author due to future publication purposes.

## Abbreviations

These are the abbreviations that are used in this manuscript:

ASL	American Sign Language
BdSL	Bangla Sign Language
SLR	Sign Language Recognition
AI	Artificial Intelligence
YOLO	You Only Look Once
RCNN	Region-based Convolutional Neural Network
AP	Average Precision
mAP	Mean Average Precision
FPS	Frames Per Second
CNN	Convolutional Neural Network
SSD	Single Shot MultiBox Detector
GFLPs	Giga Floating Point Operations Per Second
ML	Machine Learning
